# Fasting hyperglycemia upon hospital admission is associated with higher pneumonia complication rates among the elderly

**DOI:** 10.1186/1755-7682-3-16

**Published:** 2010-08-02

**Authors:** Mario R Castellanos, Anita Szerszen, Chadi Saifan, Irina Zigelboym, Georges Khoueiry, Nidal Abi Rafeh, Robert V Wetz, Morton Kleiner, Nelly Aoun, Kera F Weiserbs, Theodore Maniatis, Jeffrey Rothman

**Affiliations:** 1 Department of Medicine, Staten Island University Hospital, 450 Seaview Ave, Staten Island, NY 10305, USA; 2 Division of Geriatrics, Department of Medicine, Staten Island University Hospital, 375 Seguine Ave, Staten Island, NY 10309, USA; 3 Division of Endocrinology, Department of Medicine, Staten Island University Hospital, 450 Seaview Ave, Staten Island, NY 10305, USA; 4 Division of Pulmonary and Critical Care Medicine, Staten Island University Hospital, 450 Seaview Ave, Staten Island, NY 10305, USA

## Abstract

**Background:**

Hyperglycemia is an independent predictor of adverse outcomes during hospitalization. In patients who have pneumonia, significant hyperglycemia is associated with poor outcomes. This study evaluates the interaction of the degree of hyperglycemia and complication rates stratified by age in non-critically ill patients admitted to the hospital for care of community-acquired pneumonia.

**Methods:**

Retrospective review of patient records coded for pneumonia. Analysis included 501 non-critically ill patients admitted to a tertiary care hospital in New York City. Data were stratified by diabetes status, age (less than 65 and 65 and over), and fasting blood glucose (FBG) within the first 24 hours of hospitalization. Among patients with no history of diabetes, FBG was stratified as "normal" [FBG ≤100 mg/dl (5.6 mmol/l)], "mild-hyperglycemia" [101-125 mg/dl (5.7-6.9 mmol/l)], and "severe-hyperglycemia" [≥126 mg/dl (7 mmol/l)]. The diabetic group included known diabetics regardless of FBG. The Pneumonia Severity Index (PSI) was calculated for all patients. Complications rates, hospital length of stay and mortality were compared among the groups.

**Results:**

In patients age 65 and older, complication rates were 16.7% in normoglycemics, 27.5% in the "mild-hyperglycemia" group, 28.6% in the "severe hyperglycemia" group, and 25.5% in those with known diabetes. The mild and severe-hyperglycemics had similar complication rates (p = 0.94). Compared to the normal group, mild and severe groups had higher rates of complications, p = 0.05 and p = 0.03, respectively. PSI tended to be higher in those over the age of 65. PSI was not significantly different when the normal, mild, severe, and known diabetes groups were compared. PSI did not predict complications for new hyperglycemia (normals' mean score 87, mild 84.7, severe 93.9, diabetics 100). Hospital mortality did not differ among groups. Length of stay was longer (p = 0.05) among mild-hyperglycemics (days = 8.4 s.e. 14.3) vs. normals (days = 6.2 s.e.6.5).

**Conclusion:**

This study shows that FBS between 101-125 mg/dl (5.7-6.9 mmol/l) on hospital admission increases pneumonia complication rates among the elderly with no previous diagnosis of diabetes.

## Background

Pneumonia is the leading infectious cause of death in the elderly [1]. Annually, about 5 million Americans, mostly older adults, are diagnosed with pneumonia [2]. The presence of multiple chronic conditions, declining cough reflex, and impaired ciliary function make older individuals more susceptible to pneumonia and at increased risk for its complications [1,2].

Hyperglycemia is an independent predictor of adverse outcomes during hospitalization in multiple clinical settings, including acute myocardial infarction, stroke, and surgery [3-5]. Hospitalized individuals without a prior history of diabetes who are found to be hyperglycemic have increased mortality compared to known diabetics and those with normal glucose [6]. Hyperglycemic patients (admission blood glucose ≥ 200 mg/dl [11 mmol/l]) with community-acquired pneumonia have increased mortality and complications of pneumonia when compared to normoglycemic individuals [7].

At the molecular level, hyperglycemia affects pro-inflammatory cytokine production [8] and the function of polymorphonuclear leukocytes [9] and T cells [10]. Alterations in polymorphonuclear cells have been extensively studied, with demonstration of defects in adhesion, chemotaxis, and phagocytosis when cells are exposed to hyperglycemia [11], supporting clinically observed adverse outcomes. Observational studies associating hyperglycemia with poor patient outcome provided the basis for randomized controlled interventional studies [12-18], which have shown that treating hyperglycemia with insulin protocols improves the morbidity and mortality rates among post-operative patients in the surgical intensive care unit (ICU) and during critical illness. As a result of these studies, control of hyperglycemia during critical illness has become a standard of care. Although optimal glucose targets and protocols continue to be examined for critically ill patients, there are no interventional trials evaluating goals for glucose control in the non-intensive care setting. Observational studies found that admission glucose above 200 mg/dl (11 mmol/l) was a predictor of in-hospital complications and longer length of stay (LOS) [7]. However, there are no published studies examining whether mild hyperglycemia with fasting values between 100 mg/dl to 126 mg/dl (5.6-7 mmol/l) leads to adverse outcomes in hospitalized patients outside the intensive care setting. In this study, we evaluated patients admitted to general medical floors for the treatment of community-acquired pneumonia to determine if mild hyperglycemia affects morbidity and mortality.

## Research Design and Methods

This study was conducted at a large teaching hospital. The hospital serves a mixed urban/suburban population of nearly half a million in a borough of New York City. The medical records of patients admitted for the treatment of pneumonia during one year were examined. Patients were identified by discharge diagnosis codes for pneumonia. Eligibility criteria were: age 18 years or older, placement on a general medical floor on admission, pneumonia diagnosed by the finding of a new infiltrate on chest x-ray as documented by a radiology report with clinical symptoms suggestive of a pulmonary infection; and having an early morning fasting blood glucose (FBG) level drawn within 24 hours of admission. Patients were excluded if they were being treated for a hospital-acquired pneumonia, defined as either developing a new pneumonia 48 hours or more after admission or within 2 weeks after discharge from a hospital. Patients admitted to a critical care unit were also excluded.

Patients who met the inclusion criteria were then grouped according to their FBG level as follows: 1. "Known diabetic": patients known to have a previous history of diabetes regardless of the admission FBG, 2. "Severe hyperglycemia": a group defined by FBG of 126 mg/dl (7 mmol/l) or greater and no previous history of diabetes, 3. "Mild hyperglycemia": a group defined by FBG of 101 mg/dl to 125 mg/dl (5.7-6.9 mmol/l) and no previous history of diabetes, 4. "Normal": a group defined by FBG of less than or equal to 100 mg/dl (5.6 mmol/l).

Pneumonia complications were defined as a change from the initial admission status by development of one or more of the following during the hospitalization: "Mild" complications: 1) Increase in oxygen requirement after 24 hours of admission, 2) Increased antibiotic coverage (defined as requiring an additional antibiotic added to the initial treatment or a switch to more broad-spectrum antibiotic coverage due to worsening clinical symptoms), "Severe" complications: 1) Transfer to an intensive care unit at any time during the hospitalization, 2) Need for mechanical ventilation, 3) Development of sepsis (based upon clinical signs and symptoms of having the systemic inflammatory response syndrome), 4) Death that occurred in the course of the hospitalization was included as a severe complication. All patients were managed according to pneumonia treatment guidelines published by the American Thoracic Society [19]. The three hyperglycemic groups were compared to the normoglycemic group in terms of these main outcome variables: frequency of pneumonia complications, hospital LOS and in-hospital death rate.

Because outcomes of pneumonia are known to be worse in the geriatric population [20], patients were stratified by age (less than 65 years old and 65 years of age and above).

### Baseline Pneumonia Severity

The pneumonia severity index (PSI) [21-23] was used to stratify risk. This stratifies patients with pneumonia into five classes for the risk of death within 30 days of presentation. Predictor variables are based on the presence of co-morbidities, physical findings and selected laboratory tests, with points assigned for poor outcome variables. Blood glucose in this scoring system is considered poor if a random value is greater than 250 mg/dl (14 mmol/l).

### Statistical analysis

The effects of mild and severe hyperglycemia on pneumonia outcome were determined by examining: the frequency of pneumonia complications, LOS and in-hospital death rate. The data were collected and statistical analysis was done to determine whether patients with any level of hyperglycemia admitted for pneumonia had a longer hospital stay, higher rate of complications, or an increased mortality compared to the control patients. The data was examined using The Epic Info Statistical Package 2000. Odds ratios (OR) were calculated for each group. Continuous data are presented as mean ± SD and compared via ANOVA with 3 groups. Pearson Chi Square test was used to compare the frequency of complications within the groups. The significance level was set at p < 0.05 for all analyses.

## Results

A total of 2000 patient records were reviewed. Five hundred and one cases were included; the major reason for exclusion was the lack of a documented infiltrate on the chest x-ray report. Patients' characteristics are shown in Table 1.

**Table 1:** Characteristics of pneumonia patients admitted to general medical floors

		Admission glucose level (%)							
		**Normal** **≤100 mg/dl** **N = 56**		**Mild** **101-125 mg/dl** **N = 51**		**Severe** **>126 mg/dl** **N = 44**		**Known diabetic** **N = 37**	

**Group**		**n**	**%**	**n**	**%**	**n**	**%**	**n**	**%**

	**Average Age (se)**	50.5 (10.8)	-	46.7(13.5)	-	51 (10)	10.0	46.2 (10.4)	-

**18-64**	**Gender**								
	
	Male	30	53.6	30	58.8	21	47.7	24	64.9
	
	Female	26	46.4	21	41.2	23	52.3	13	35.1
	
	History of COPD*	4	7.3	6	11.8	^¶¶¶^**18**	40.9	7	18.9
	
	**Heart Failure**	8	14.3	^¶^**1**	2.0	2	4.6	2	5.4
	
	**Liver disease**	6	10.7	2	3.9	3	6.8	4	10.8
	
	**Cancer**	5	8.9	3	5.9	4	9.1	2	5.4
	
	**Chronic renal disease**	10	17.9	4	7.8	3	6.8	9	24.3
	
	**Mean PSI^†^**	61.4 (se 4.1)		61.6 (se 4.1)		61.3 (se 4.4)		74.4 (se 4.2)	

									

		**N = 69**		**N = 68**		**N = 99**		**N = 77**	

		**n**	**%**	**n**	**%**	**n**	**%**	**n**	**%**

	**Average Age (se)**	78.2 (7.3)		80.9 (9.1)		80.6(8.6)		80.0 (7.4)	

**65 and Above**	**Gender**								
	
	Male	36	52.2	^¶^**24**	35.8	48	48.5	43	55.8
	
	Female	33	47.8	43	64.2	51	51.5	34	44.2
	
	**Living arrangement**								
	
	**Community**	54	78.3	53	77.9	73	73.7	58	75.3
	
	**Nursing home**	15	21.7	5	22.1	26	26.3	19	24.7
	
	**History of COPD^†^**	19	27.5	19	28.0	30	30.6	14	18.2
	
	**Heart Failure**	18	26.1	10	14.7	^¶^**13**	13.1	22	28.6
	
	**Liver disease**	1	1.5	3	4.4	4	4.0	2	2.6
	
	**Cancer**	6	8.7	7	10.5	12	12.1	10	13.0
	
	**Chronic renal disease**	24	34.8	17	25.0	22	22.2	19	24.7
	
	PSI (mean se) *	107.8 (se = 4.5)		102.1 (se = 3.5)		108.4 (se = 3.4)		112.4 (se = 3.1)	

^†^COPD, chronic obstructive pulmonary disease

*Pneumonia Severity Index (mean and standard error), t-test compares each group with the normal group (admission glucose ≤99 mg/dl), *p ≤ 0.05

^¶^χ^2 ^tests compares each group with the normal group (admission glucose ≤99 mg/dl) ^¶^0.01<p ≤ 0.05, ^¶¶^0.001≤p ≤ 0.01, ^¶¶¶^p ≤ 0.001

Note: Highlighted values were statistically different from the normal group; all others were not statistically significant

In the under 65 years of age group the mean age was not significantly different among all the glucose groups and ranged between 46.2 to 51 years. In this age set, the mild-hyperglycemia group (N = 51) had fewer patients with congestive heart failure (CHF). It was otherwise comparable to the normal group (N = 56) with respect to all other comorbidities including average PSI. The severe hyperglycemia group (N = 44) had more patients with chronic obstructive pulmonary disease (COPD) compared to normals. They were otherwise similar to the normal group with respect to all other characteristics including PSI. Finally, in the under age of 65 group, the known diabetics (N = 37) were statistically similar to the normal controls in all baseline characteristics.

In the older patients group, aged 65 and over, the mean age range was 78.2-80.9 among all the glucose groups. In this age group, the mild-hyperglycemia group (N = 68) had significantly fewer men. It was otherwise comparable to the normal glucose group (N = 69) in all aspects, including comorbidities and average PSI. The severe hyperglycemia group (N = 99) had significantly fewer patients with CHF compared to the normals. However, their other characteristics were statistically similar to those of the normals, including PSI. Finally, the known diabetics in the 65 and above group (N = 77) were statistically similar to the controls in all aspects, comorbidities and PSI.

The average PSI scores and the more specific PSI classes are listed in Table 2. In both age groups, all the hyperglycemia patients (mild, severe, and known diabetics) had similar numbers of patients in each PSI class and did not differ statistically. As expected, the older patients had higher PSI scores compared to the younger patients.

**Table 2:** Pneumonia severity index stratified by admission glucose level

			Admission glucose level (%)							
**Age**	**Pneumonia Severity Index**		**Normal** **≤100 mg/dl** **N = 56**		**Mild** **101-125 mg/dl** **N = 51**		**Severe** **>126 mg/dl** **N = 44**		**Known** **diabetic** **N = 37**	

**18-64**	**Class**	Points	N	%	N	%	N	%	N	%
	
	**II**	≤70	37	66.1	35	68.6	32	72.7	19	51.4
	
	**III**	71-90	8	14.3	8	15.7	8	18.2	8	21.6
	
	**IV**	91-130	9	16.1	6	11.8	3	6.8	9	24.3
	
	**V**	>130	2		2	3.9	1	2.3	1	2.7

										

			**N = 69**		**N = 68**		**N = 99**		**N = 77**	

	**Class**	Points	N	%	N	%	N	%	N	%

**65+**	**II**	≤70	7	10.1	8	11.8	9	9.1	3	3.9

	**III**	71-90	16	23.2	17	25.0	28	28.3	17	22.1

	**IV**	91-130	32	46.4	31	45.6	33	33.3	37	48.1

	**V**	>130	14	20.3	12	17.7	29	29.3	20	26.0

*χ^2 ^compares each group with the normal group (admission glucose ≤99 mg/d) * p ≤ 0.05

Note: No significant differences in PSI class among the glucose groups.

In the younger patients, the development of pneumonia complications did not differ in any of the hyperglycemia groups compared to the normal (Table 3). Similarly, the number of deaths and hospital LOS in all these groups did not differ significantly.

**Table 3:** Pneumonia complications by admission glucose level among adults aged 64 and below^‡^

	Admission glucose level (%)												
	**Normal ≤100 mg/dl** **N = 56**	**Mild 101-125 mg/dl** **N = 51**				**Severe >126 mg/dl** **N = 44**				**Known diabetic** **N = 37**			

	%	**%**	**OR**	**95% CI**	**p**	**%**	**OR**	**95% CI**	**p**	**%**	**OR**	**95% CI**	**p**

Complications Types*													

↑ O_2 _requirements	17.7	19.6	1.12	0.42-0.82	0.82	30	1.92	0.75-0.17	0.17	24.3	1.47	0.57-4.08	0.45

↑ABx coverage	23.2	17.7	0.71	0.27-1.83	0.47	25	1.03	0.44-2.77	0.8	16.2	0.64	0.21-1.87	0.41

Sepsis	12.5	5.7	1.30	0.44-3.89	0.64	13.7	1.05	0.34-3.56	0.67	18.9	1.63	0.52-5.12	0.4

Subsequent ICU use	10.9	11.8	1.09	0.33-3.62	0.9	13.7	1.29	0.39-4.32	0.67	18.9	1.91	0.58-6.21	0.4

Mechanical-vent	14.3	11.8	0.80	0.26-0.70	0.7	11.4	0.77	0.23-2.54	0.67	18.9	1.40	0.46-4.26	0.55

Deaths (N)	3.6 (2)	2(1)	0.54	0.48-0.62	0.6142	6.8(3)	1.98	0.32-12.37	0.46	0	--	--	0.2516

													

**Category**													

No complications	80.0	80.0	--	--	--	75.0	--	--	--	75.7	--	--	--

Mild*	1.8	4.7	2.20	0.19-25.12	0.52	5.7	2.73	0.23-31.56	0.40	0	--	--	--

Severe^†^	18.8	16.3	0.88	0.32-2.45	0.80	21.4	1.23	0.45-3.36	0.69	24.3	1.44	0.52-4.00	0.47

Any one complication	52.4	19.6	1.00	0.38-2.60	1.00	25.0	1.36	0.53-3.52	0.52	24.3	1.33	0.48-3.57	0.59

Any two complications	53.3	14.6	0.96	0.32-2.88	0.94	17.5	1.19	0.39-3.61	0.75	20.0	1.43	0.46-4.30	0.54

													

Total admission days (mean se)^¶^	6.0 se = 5.9	10.6 se = 21.0				7.6 se = 6.6				6.1 se = 4.5			

**Age **(mean se)	50.5 se = 10.8	46.7 se = 3.5				51.0 se = 10.0				46.2 se = 10.4			

*Mild complications: either ↑ O_2 _requirements, or ↑ Antibiotic coverage

^†^Severe complications: either subsequent ICU use, mechanical ventilation, sepsis and/or death.

^‡ ^χ^2 ^compares each glucose category with the reference group (normal ≤99 mg/dl).

The pneumonia complication rate varied significantly by admission glucose in the elderly patients (age 65 and over) (Table 4). In the elderly, the severe hyperglycemia group consisted of 99 patients. This group had a significantly increased risk of developing any one complication (39.4%, p = 0.03) and any two complications (33.3%, p = 0.06) during the hospitalization when compared to the normal group (Table 4). The mild hyperglycemia group consisted of 68 patients. This group had a statistically significant risk of developing any one or two complications compared to the normal group (39.7%, p = 0.04 and 37.9%, p = 0.02 respectively). The elderly patients with known diabetes (N = 77) did not have a statistically significant increased rate of developing any one or any two complications compared to the normal group (35.1%, p = 0.12 and 29.6%, p = 0.18 respectively).

**Table 4:** Pneumonia complications by admission glucose adults aged 65 and above

	Admission glucose level (%)												
	Normal ≤100 mg/dl N = 69	Mild 101-125 mg/dl N = 68				Severe >126 mg/dl N = 99				Known diabetic N = 77			
Complications Types*	**%**	**%**	**OR**	**95% CI**	**p**	**%**	**OR**	**95% CI**	**p**	**%**	**OR**	**95% CI**	**p**

													

↑ O_2 _requirements	21.7	41.2	2.52	1.19-5.32	**0.014**	44.4	2.88	1.44-5.78	**0.002**	35.1	1.94	0.92-4.07	0.075

↑ ABx coverage	23.5	32.4	1.55	0.73-3.31	0.25	28.3	1.28	0.63-2.61	0.49	28.6	1.30	0.62-2.74	0.49

Sepsis	14.5	26.5	2.12	0.90-5.01	0.08	23.2	1.79	0.79-4.04	0.16	23.4	1.80	0.77-4.22	0.17

Subsequent ICU use	11.6	22.1	2.16	0.85-5.49	0.10	16.2	1.47	0.59-3.65	0.41	16.9	1.55	0.60-4.00	0.36

Mechanical-vent	10.1	19.1	2.09	0.78-5.62	0.1370	14.1	1.45	0.56-3.83	0.44	11.7	1.17	0.41-3.34	0.77

Deaths (N)	10.3 (7)	12 (8)	1.17	0.40-3.40	0.78	12(12)	1.22	0.45-3.27	0.69	9 (7)	0.88	0.29-2.66	0.8

													

Category													

No complications	76.5	60.3	--	--	--	59.8				64.9	--	--	--

Mild*	20.9	4.4	1.94	0.31-12.15	0.47	10.3	2.73	0.24-31.36	**0.40**	11.4	4.77	0.98-23.16	**0.03**

Severe^†^	20.6	35.3	2.22	1.02-4.81	0.04	29.9	1.83	0.88-3.82	**0.11**	23.4	1.36	0.31-3.03	0.45

Any one complication	23.5	39.7	2.19	1.04-4.58	**0.04**	40.2	2.15	1.08-4.29	**0.03**	35.1	1.79	0.86-3.71	0.12

Any two complications	20.0	37.9	2.49	1.13-5.45	**0.02**	34.1	2.04	0.96-4.31	**0.06**	29.6	1.71	0.78-3.79	0.18

**Age **(mean se)	78.2 se = 7.3	80.9 se = 9.1				80.6 se = 8.6				80.0 se = 7.4			

**Total admission days** (mean se)^¶^	6.3 se = 6.0	6.7 se = 4.7				7.7 se = 7.0				7.3 se = 7.3			

*Mild complications: either ↑ O_2 _requirements, or ↑ Antibiotic coverage

^†^Severe complications: either subsequent ICU use, mechanical ventilation, sepsis and/or death

^‡^χ^2 ^compares each group with the reference group (admission glucose ≤99 mg/dl).

Note: Highlighted values were statistically different from the normal group; all others were not statistically significant

The in-hospital mortality rate of the elderly was also assessed and did not vary significantly among the different groups (Table 4). The LOS or total admission days among the groups, which include those with and without complications, was not significantly different. The mean LOS for the severe hyperglycemia group was 7.7 +/- 7.0 days; the mild hyperglycemia group had a LOS of 6.7 +/- 4.7 days; the diabetics had a LOS of 7.3 +/- 7.3 and the control group a LOS of 6.3 +/- 6.0.

## Discussion

This study compared pneumonia-related complication rates among hospitalized, non-critically ill patients with elevated blood glucose levels in the range of 101 to 125 mg/dl (5.7-6.9 mmol/l) to those with overt fasting hyperglycemia of ≥126 mg/dl (7 mmol/l) as well as to patients with known diabetes and those with normal glucose values. We chose to study the effects of hyperglycemia among patients with pneumonia since pneumonia is a common cause for hospitalization in the elderly and the adverse effects of hyperglycemia may potentially contribute to increased complication rates [24].

Patients were stratified into two distinct age groups and by PSI class to identify those who may be at higher risk for complications on admission. Among our 501 patients the prevalence of diabetic range hyperglycemia was 28.5% (in patients without a history of diabetes). The rate of known diabetes was 22% and the range of patients with mild hyperglycemia on admission was 24%. In our study there were no differences in pneumonia complication rates in younger patients (< 65 years) when assessed by admission glucose. Among the elderly, newly diagnosed fasting mild hyperglycemia was associated with a higher rate of pneumonia complications compared to those with a normal glucose level on admission.

This study demonstrates that even mild elevation of glucose, between 101 and 125 mg/dl (5.7-6.9 mmol/l), in an elderly group of patients with no history of diabetes is associated with an increase in the pneumonia complication rates. The OR for developing any one complication was 2.2 (1.04-4.58) in the elderly mild hyperglycemia group compared to normoglycemic patients. This was similar to patients with overt diabetic range hyperglycemia, who had an OR of 2.15 (1.08-4.29) (Table 4). The PSI on admission was not statistically different among all the groups and did not predict hospital complications or identify those that would subsequently require ICU admission. Although initially not developed for this purpose, clinicians use PSI to risk-stratify patients on admission. Given current evidence, a glucose value of 250 mg/dl (14 mmol/l) as a part of the risk assessment seems too high [7].

Neither hospital mortality nor LOS was statistically different among any of the groups, possibly due to the relatively small sample size. To determine the sample size to study these variables the prevalence of mild hyperglycemia among non-critically ill hospitalized patients needed to be established, our study provides this data. In addition, our results confirm evidence provided by McAlister et al [7] that admission hyperglycemia in the diabetic range is associated with adverse outcomes in elderly patients with pneumonia. In that study, the median age of patients was 75 years. The current study further documents that even mild hyperglycemia consistent with pre-diabetes is related to higher complication rates in patients over the age of 65.

Our findings provide the information to conduct future research. Admission hyperglycemia, even in the non-diabetic range, may be a marker of immune dysfunction and/or a pro-inflammatory state and should aid in the identification of patients at higher risk for complication rates during hospitalization. In this study, 50% of elderly patients who developed pneumonia had newly diagnosed, apparently stressed-induced, hyperglycemia. This puts forward the need for more aggressive screening of all hospitalized patients. Our study suggests that fasting glucose levels previously considered not to warrant intervention may be associated with deleterious effects. Further studies should be conducted to evaluate this possibility.

## Competing interests

The authors declare that they have no competing interests.

## Authors' contributions

MC: study concept and design; data interpretation manuscript preparation, AS: data management and interpretation, manuscript preparation, CS: data collection and management, IZ: data collection and interpretation, GK: data collection, NAF: data collection, RW: data interpretation, manuscript preparation, MK: manuscript preparation, NA: data collection, KW: statystical analysis and data interpretation, TM: study design and coordination, manuscript review, JR: study concept and design, data interpretation, manuscript preparation.

All authors read and approved the final manuscript.
